# Supplementary material for: Sorting at embryonic boundaries requires high heterotypic interfacial tension
Source: Nat Commun. 2017 Jul 31;8:157. doi: 10.1038/s41467-017-00146-x (PMC5537356; doi:10.1038/s41467-017-00146-x)
Supplement: Supplementary file 2 — Supplementary Software 1 [file 41467_2017_146_MOESM2_ESM.zip › PottsModel/SrcPottsModel/doc/simulations/package-frame.html]

simulations


# simulations

## Classes

- BoundarySimulation
- DefaultSimulation
- DITHSimulation
- EndoSimulation
- FourByFourSimulation
- GenerateRandomLattices
- GranerGlazierSimulation
- NegativeControl
- SingleCell
